# Original Translations

**Published:** 1882-03

**Authors:** 


### Original Translations.

ETIOLOGY AND PROPHYLAXIS OF TUBERCULOSIS. By AUSTIN C. RAMSAY, Chicago.

Tuberculosis is a contagious, infectious, parasitic, non-hereditary disease. This will surely find many skeptics, but such is the view admitted to-day, by many eminent physicians in France and abroad, and is taught at the Paris Medical Faculty, by Dr. Bouchard, professor of general pathology. This view of the matter is destined to make a great revolution in the therapeutics of tuberculosis. We will call attention here to the considerations on which the new doctrine is based, and to the objections which it suggests, then we will look for the practical consequences resulting therefrom.

The most important argument in favor of the contagiousness of tuberculosis, lies in the possibility of transmitting the disease by the inoculation, or ingestion, of tuberculous matter. Although the infectious agent, the tubercular germ, be yet unknown (though Klebs and Eklund thought they discovered in the expectorations and in the lung cavities of the tubercular patients a special microphyte) the possibility of positive inoculation is none the less demonstrated.

To Dr. Villemin's experiments, the results of which were received with scepticism, soon were added Toussaint's experiments. He inoculated into hogs lymph taken from the muscles of tuberculous cows, and blood from phthisical soldiers, and started in the hogs, which are but slightly liable to the disease, well-marked tuberculosis.

The same results were obtained on dogs and rabbits. In the human species, the tubercles are transmissible by inoculation—at least it seems to have resulted so from experiments made on man by physicians who, happily, are not countrymen of ours. For, not the peculiar circumstances under which they operated, nor the aim in view, would justify such harshness. The contagiousness of tuberculosis finds another important support in cases of acute granular phthisis, which affects the clinical features of infectious fevers, and are remarkable from an anatomo-pathological point of view, by the general spread of deposit.

Tuberculosis is a disease no more confined to man than charbon. It is rather the chief disease of milch cows, especially those kept in stables, which are all naturally tuberculous. Other species of animals are more liable to tuberculosis than man; rabbits, for instance, never fail contracting the disease by inoculation. The number of consumptives, it is true, is great, but it is surprising that man,—living where the microphytes abound, *quœrentes quem devorent*, living on the milk and flesh of tuberculous cows, inhaling air infected with the dried expectorations of consumptives,—does not pay a larger tribute to that dreadful disease. The cause of this is that tuberculosis does not always find a congenial soil. But let the human organism be poorly protected, let it be prepared by a diathetic disease,—scrofula, for example, long convalescence, poor hygienic surroundings, physical or mental overwork, poor nourishment, moist climate, impure atmosphere,—in a word, let the microphytes find a poorly protected organism, their receptibility is created, their victory certain and their development most always fatal. But if we admit the contagious nature of the tuberculosis, what becomes of the principle, the clinical dogma, of the hereditary transmission of that disease? This is the weak point of the new theory, and the principal objection against it. Dr. Bouchard has well foreseen it. It is not the contagious disease which is transmitted to the offspring, it is the constitution which remains the same in the father and the son. The child of a man broken down by phthisis, will necessarily be a debilitated being, unable to resist the tubercular infection. The causes of tuberculosis may be divided into two: (*a*) the immediate determining and predisposing cause, residing in the infectious agent; (*b*) the occasional and predisposing cause residing in the morbid tendencies. The prophylaxis of tuberculosis equally comprises two indications: (*a*) defend the predisposed individuals by hygienic precautions; (*b*) modify the habits of life, change the organic surroundings.

The first point is merely an addition to the ordinary prescriptions of the physician, yet a stranger to the new doctrine (sea voyages, fresh air, avoiding of excesses, etc.) Another recommendation which will seem too severe, and impossible: The careful supervision of the diet of the predisposed to tuberculosis, "the careful exclusion of any milk or meat which may contain tuberculous matter." Since all cows in stabling are tuberculous, this means a complete prohibition of milk and meat, at least in regard to the poorer classes in cities. Who will dare proclaim this, which would prove an arrest of death for so many new-born children?

As to the second point, the aim in view will be attained by living in the open air, by providing for plenty of good food, and taking the proper care of individuals. A beneficial result may also follow sulphur and salt-water baths, which are of a nature to strengthen the constitution, and increase its resisting powers. —*Revue Médicale*, July 2, 1881.

---

PROFESSOR PIROGOFF, whose death was lately announced, had for many years withdrawn from the active practice of his profession, and had lived on his estate in the country, where he gave his valuable advice gratuitously to the numerous patients who flocked to him from the surrounding districts. In America his name is chiefly known in connection with an amputation at the ankle-joint, in which the tuberosity of the os calcis is left in the heel flap. He was the author of a Medical History of the Crimean War, and of treatises on Orthopædic Surgery, the Surgery of Arteries, and an Atlas of Topographical Anatomy. He was the principal medical officer of the General Hospital in Sebastopol during the Crimean war, and took an active part in the introduction of plaster-of-Paris bandages.

